# Rheumatological Manifestations in People Living with Human T-Lymphotropic Viruses 1 and 2 (HTLV-1 and HTLV-2) in Northern Brazil

**DOI:** 10.3390/v17070874

**Published:** 2025-06-20

**Authors:** Márcio Yutaka Tsukimata, Bianca Lumi Inomata da Silva, Leonn Mendes Soares Pereira, Bruno José Sarmento Botelho, Luciana Cristina Coelho Santos, Carlos David Araújo Bichara, Gabriel dos Santos Pereira Neto, Aline Cecy Rocha Lima, Francisco Erivan da Cunha Rodrigues, Natália Pinheiro André, Sarah Marques Galdino, Danniele Chagas Monteiro, Ludmila do Carmo de Souza Silva, Lourena Camila Oliveira Araújo, José Ronaldo Matos Carneiro, Rosana de Britto Pereira Cruz, Ricardo Ishak, Antonio Carlos Rosário Vallinoto, Bárbara Nascimento de Carvalho Klemz, Izaura Maria Vieira Cayres Vallinoto

**Affiliations:** 1 Laboratory of Virology, Institute of Biological Sciences, Federal University of Pará, Belém 66075-110, Pará, Brazil; marcio.tsukimata@ics.ufpa.br (M.Y.T.); inomatabianca@gmail.com (B.L.I.d.S.); leonnpereira@hotmail.com (L.M.S.P.); bruno.botelho@ics.ufpa.br (B.J.S.B.); lccp.santos27@gmail.com (L.C.C.S.); bichara@amaralcosta.com.br (C.D.A.B.); gabrielnetoenf@gmail.com (G.d.S.P.N.); alinececy@yahoo.com (A.C.R.L.); erivan218@gmail.com (F.E.d.C.R.); natalia.andre@ics.ufpa.br (N.P.A.); sarahmarques15@gmail.com (S.M.G.); danniele.monteiro@ics.ufpa.br (D.C.M.); ludmiladesousa2019@gmail.com (L.d.C.d.S.S.); lourena.araujo@ics.ufpa.br (L.C.O.A.); rishak@ufpa.br (R.I.); vallinoto@ufpa.br (A.C.R.V.); 2 Faculdade de Medicina, Instituto de Ciências Médica, Universidade Federal do Pará, Belém 66075-110, Pará, Brazil; ronaldomatos@ufpa.br (J.R.M.C.); rbpcruz@ufpa.br (R.d.B.P.C.); 3 Programa de Pós-Graduação em Biologia de Agentes Infecciosos e Parasitários, Universidade Federal do Pará, Belém 66075-110, Pará, Brazil; 4 Amaral Costa Medicina Diagnóstica, Belém 66055-050, Pará, Brazil

**Keywords:** human T-lymphotropic virus 1 (HTLV-1), human T-lymphotropic virus 2 (HTLV-2), rheumatic diseases, arthralgia, myalgia

## Abstract

Human T-lymphotropic virus 1 (HTLV-1) infection has been associated with inflammatory, autoimmune, and lymphoproliferative diseases with a wide spectrum of clinical manifestations. Among patients with inflammatory rheumatological disease manifestations, cases of rheumatoid arthritis, Sjögren’s syndrome, polymyositis, and fibromyalgia, among others, have been reported. Another common feature of rheumatological diseases is the presence of joint manifestations, such as arthralgia and arthritis. In the present study, we sought to determine the laboratory profile and clinical rheumatological manifestations of people living with HTLV-1/2 residing in a metropolitan area in the Brazilian Amazon. A total of 957 individuals were screened for HTLV-1/2 infection by enzyme-linked immunosorbent assay (ELISA), and samples from seropositive individuals were subjected to infection confirmation by Western blotting or quantitative polymerase chain reaction (qPCR). Individuals with confirmed HTLV-1 and HTLV-2 infection were clinically evaluated for signs and symptoms of rheumatological diseases. Of the 957 individuals tested, 69 were positive for HTLV-1/2 infection, with 56 confirmed cases of HTLV-1 infection (5.9%), 12 of HTLV-2 infection (1.2%), and 1 classified as undetermined (0.1%). After clinical screening, 15 infected individuals with complaints suggestive of rheumatological disease were selected for evaluation by a rheumatologist (11 with HTLV-1 infection (1.1%) and 4 with HTLV-2 infection (0.4%)). The predominant pain pattern was symmetrical polyarthralgia, with large joints predominantly being affected. The diseases diagnosed were psoriatic arthritis, osteoarthritis, fibromyalgia, and regional pain syndromes. Antinuclear antibody (ANA) positivity was observed in two patients. Our findings confirm that HTLV-1 infection is associated with rheumatological disease manifestations and highlight the novel finding of cases of HTLV-2 infection in patients with rheumatoid arthritis symptoms.

## 1. Introduction

Human T-lymphotropic virus (HTLV) belongs to the *Retroviridae* family, genus *Deltaretrovirus* [1]. HTLV is classified into four types, HTLV-1, HTLV-2, HTLV-3, and HTLV-4, with HTLV-1 and HTLV-2 being the most widespread and being present in several regions of the world, especially in Japan, the Caribbean, South America, Australia, and Central Africa [2].

HTLV-1 was discovered in 1980 when it was isolated from a patient with cutaneous T-cell lymphoma [3]. In 1982, HTLV-2 was isolated from a patient with hairy cell leukemia [4]. HTLV-3 and HTLV-4 were more recently identified as infecting nonhuman primate hunters living in isolated forests in the Republic of Cameroon but have not been associated with clinical manifestations to date [5,6].

It is estimated that at least 10% of people living with HTLV-1/2 (PLWH) may develop symptoms of diseases associated with the infection [7], especially adult T-cell leukemia/lymphoma (ATLL) and HTLV-associated myelopathy/tropical spastic paraparesis (HAM/TSP) [8,9]. However, HTLV-1 infection has been associated with respiratory, rheumatological, dermatological, and ophthalmological diseases [10,11,12,13]. HTLV-2, in turn, has been associated less frequently with cases of lung disease, neurological disease similar to HAM/TSP [14,15,16], and more recently, fibromyalgia [17].

Some epidemiological studies have revealed that the prevalence of HTLV-1 infection is greater in patients with rheumatological diseases (e.g., rheumatoid arthritis, Sjögren’s syndrome, and polymyositis) than in healthy individuals [11,18,19,20,21,22,23]. Other studies have demonstrated the occurrence of clinical joint manifestations such as arthralgia and arthritis in people living with HTLV-1 [11,24,25]. However, broader and more detailed studies are required to determine the association of HTLV-1 infection with rheumatological manifestations [26,27,28].

The first study showing the relationship between HTLV-1 infection and the occurrence of rheumatological manifestations took place in Japan in 1988, when the first case report of a patient with HTLV-1 presenting with arthritis appeared [29]. In the same year, in the West Indies, five patients with HTLV-1 diagnosed with Sjögren’s syndrome were described [30]. Since then, findings from several studies have suggested a relationship between infection by a virus and the development of rheumatological diseases, mainly inflammatory autoimmune diseases [26,27,28].

In addition to these epidemiological findings that suggest a clinical correlation between HTLV-1 infection and the development of rheumatological diseases, the presence of the virus was found in biopsy samples of synovial tissue from infected patients [31]. In experiments with mice transgenic for the HTLV-1 *tax* and *hbz* genes, the manifestation of arthritis and other inflammatory diseases was observed, suggesting that these viral proteins are related to the pathogenesis of HTLV-1-associated arthritis [32].

In search of a better understanding of the relationship between the presence of HTLV-1 and HTLV-2 infection and the clinical manifestations of rheumatological diseases, the aim of the present study was to determine the sociodemographic and laboratory profiles and the occurrence of rheumatological manifestations in PLWH treated at a specialty service for the diagnosis and monitoring of PLWH residing in the city of Belém, the capital of the state of Pará, one of the three Brazilian states with the highest prevalence of HTLV-1 and HTLV-2 infections [33,34].

## 2. Materials and Methods

### 2.1. Study Design and Population

For this descriptive clinical–laboratory cross-sectional study, a quantitative approach was used to recruit users of a service provided by the Virology Laboratory (LabVir) of the Federal University of Pará (UFPA) called the Service for Assistance to People Living with HTLV (SAPEVH), which includes monitoring by a rheumatologist.

The study was conducted with individuals who received a confirmatory diagnosis of HTLV-1 or HTLV-2 infection. These individuals were identified (i) by recruiting at health activities promoted by LabVir in several locations in the metropolitan region of Belém (primary health unit, churches, and community centers); (ii) by searching for individuals with a confirmed diagnosis and positive serological screening results at a blood bank; and (iii) by recruiting individuals who had information about the service (SAPEVH) and spontaneously sought out the laboratory to undergo testing. The patients in this study were evaluated from April 2022 to March 2024. During this period, 957 individuals were investigated for infection, and those with a confirmed diagnosis of HTLV-1 or HTLV-2 infection were referred for medical evaluation at the SAPEVH outpatient clinic.

### 2.2. Ethical Considerations

This study was reviewed and approved by the Human Research Ethics Committee of the Health Sciences Institute of the Federal University of Pará (CAAE: 27290619.2.0000.0018 and CAAE: 71261523.1.0000.0018) in accordance with the guidelines of the Declaration of Helsinki. Informed consent was obtained from the patients for the publication of this article.

### 2.3. HTLV Diagnosis

Plasma measurement of total anti-HTLV-1/2 antibody titers was performed via enzyme-linked immunosorbent assay (ELISA) (Murex HTLV-I+II, DiaSorin, Dartford, UK) following the manufacturer’s protocol. Samples with reactive or indeterminate results (cutoff = 0.284) were subjected to confirmatory Western blot (HTLV Blot 2.4 kit, MP Diagnostics, Singapore) and/or quantitative polymerase chain reaction (qPCR) with the TaqMan system (Applied Biosystems, Foster City, CA, USA) on the Applied Biosystems StepOne Plus Real-Time PCR platform, following a previously described protocol [34].

### 2.4. Clinical Analysis

All individuals with a confirmed diagnosis of the infection were included in SAPEVH and began to receive counseling and clinical care from the team of nurses and doctors. During the initial anamnesis recording by nursing and general practitioners when complaints arose and clinical signs suggestive of diseases associated with HTLV-1 were present, patients were referred to medical specialists, as proposed by the Ministry of Health [35]. Patients who presented with rheumatological complaints were evaluated according to standard medical procedures [36]. Psoriatic arthritis was investigated based on the presence of 3 criteria (current psoriasis, nail dystrophy, and dactylitis) according to the Classification Criteria for Psoriatic Arthritis (CASPAR) criteria [37].

### 2.5. Autoantibody Testing

To screen for inflammatory autoimmune diseases, all patients were initially asked to undergo antinuclear antibody (ANA) testing. ANA testing was performed via indirect immunofluorescence using the Titerplane IIFT technique: HEp-2 (Euroimmun, Lübeck, Germany). For the reactive samples, the immunofluorescence pattern and titers were evaluated. For patients with joint manifestations with characteristics more related to inflammatory autoimmune diseases, testing for more specific autoantibodies, such as rheumatoid factor and anti-cyclic citrullinated protein (anti-CCP), was requested. Since these tests were temporarily unavailable at LabVir, patients were advised, if possible, to undergo tests at other laboratories in the public or private network.

## 3. Results

Among the 957 individuals tested, 69 were identified as positive for HTLV infection, with 56 confirmed cases of HTLV-1 infection (5.9%), 12 of HTLV-2 infection (1.2%), and 1 case classified as undetermined (0.1%). After clinical screening, 15 infected individuals who presented complaints suggestive of rheumatological diseases were selected for evaluation by a rheumatologist, with 11 diagnosed with HTLV-1 infection (1.1%) and 4 with HTLV-2 infection (0.4%). The sociodemographic data of the people treated at the SAPEVH rheumatology outpatient clinic are presented in Table 1. Among the 15 patients, 12 were female (9 with HTLV-1 infection and 3 with HTLV-2 infection), and 3 were male (2 with HTLV-1 infection and 1 with HTLV-2 infection), with ages ranging between 30 and 81 years and a mean age of 53.4 years (51 years for patients with HTLV-1 infection and 60 years for patients with HTLV-2 infection). In terms of race/ethnicity (skin color), most of the patients were brown.

The main joint manifestation found in the patients studied was the complaint of arthralgia, with 13 cases of this condition reported (10 with HTLV-1 infection [76.9%] and 3 with HTLV-2 infection [23.1%]). Two cases of arthritis were observed in HTLV-1 patients. Each patient with arthralgia or arthritis was classified according to the number of joints affected. The frequency of the affectation of these peripheral joints was calculated according to location (hands, wrists, elbows, shoulders, feet, ankles, hips, and knees) and is presented in Table 2.

Muscle pain was classified according to topography; the data are shown in Table 3. In total, there were eight patients with myalgia (five with HTLV-1 infection [62.5%] and three with HTLV-2 infection [37.5%]). Four individuals presented with localized pain (three with HTLV-1 infection and one with HTLV-2 infection), and four presented with diffuse pain in the body (two with HTLV-1 infection and two with HTLV-2 infection).

For the four patients with diffuse muscle pain in the body (two with HTLV-1 infection and two with HTLV-2 infection), other clinical characteristics suggestive of fibromyalgia (chronic pain, tender points, sleep disorders, and mood disorders) were evaluated and were present in all individuals assessed (Table 3), as previously described [17].

**Figure 1:**
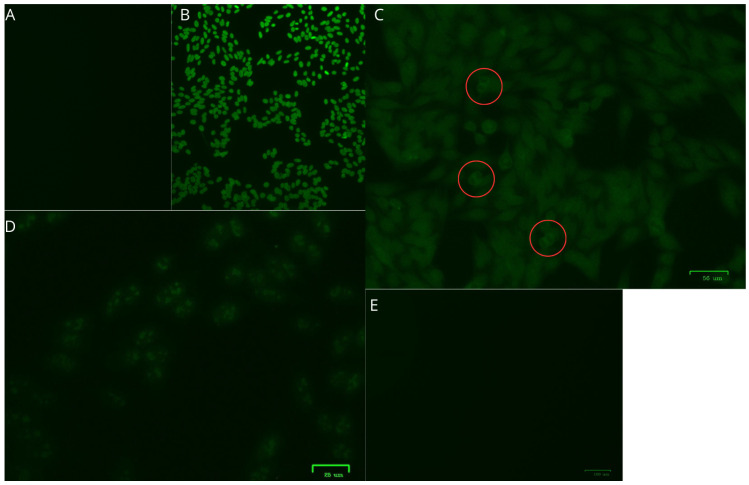
Qualification and titration of antinuclear antibody (ANA) in Patient 1: (**A**) Negative control. (**B**) Positive control. (**C**) A 1:10 dilution showing weak fluorescence and probable metaphase plates (circled in red). (**D**) A 1:100 dilution showing weak fluorescence and a probable nucleolar pattern. (**E**) A 1:1000 dilution showing negative fluorescence was used to determine the titration.

**Figure 2:**
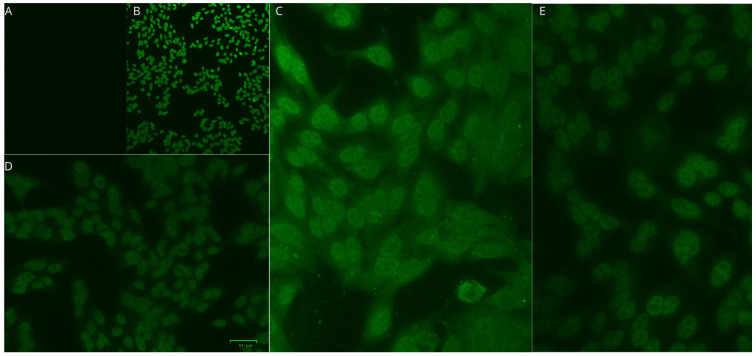
Qualification and titration of antinuclear antibody (ANA) in Patient 2: (**A**) Negative control. (**B**) Positive control. (**C**) A 1:10 dilution showing strong fluorescence with probable characteristics of a coarse speckled nuclear pattern. (**D**) A 1:100 dilution with clear fluorescence. (**E**) A 1:10,000 dilution with preservation of fluorescence and indicative of a high antibody titer greater than 1:10,000.

The main diagnoses of rheumatological diseases observed in the 15 patients infected with HTLV-1/2 are presented in Table 4. In general, 86.7% of the samples were nonreactive to ANA, rheumatoid factor, and anti-CCP testing. Among the patients with reactive samples, two were infected with HTLV-1. One sample exhibited a fine speckled nuclear immunofluorescence pattern with a titer of 1:3200, and the other sample showed a homogeneous nucleolar pattern with a titer of 1:320 (Figure 1 and Figure 2). Among the samples tested for rheumatoid factor and anti-CCP, there were no reactive samples.

## 4. Discussion

In the present study, we report the occurrence of rheumatological manifestations in PLWH residing in the city of Belém, the capital of the state of Pará, one of the three Brazilian states with the highest prevalence of HTLV-1 and HTLV-2 infections [33,34]. Our results show rheumatological manifestations in individuals infected with HTLV-2.

HTLV-1 infection can result in several clinical manifestations of inflammatory origin, with neurological disorders related to HAM/TSP being the most commonly described [38,39]. However, the clinical manifestations associated with the virus, when they arise, extend to other medical specialties, such as rheumatology, and several cases of arthritis, myositis, and arthralgia have been reported in HTLV-1 carriers; furthermore, a specific term has even been created, HTLV-1-associated arthropathy, as an attempt to standardize these rheumatological characteristics in people living with HTLV-1 [31]. However, there is still no consensus on the standardization of this arthropathy, which is sometimes classified as monoarticular or oligoarticular and sometimes as symmetrical polyarthralgia [11,26,27]. Another drawback to the term “HTLV-1-associated arthropathy” is the non-inclusion of individuals infected with HTLV-2; however, until the present study, there was no information on rheumatological manifestations in individuals infected with this HTLV type.

In the present study, a high prevalence of arthralgia was identified in individuals infected with HTLV-1/2, but only two cases of arthritis (monoarthritis and symmetrical polyarthritis) were observed, and arthritis was exclusive to HTLV-1 carriers. If patients with HTLV-1 and HTLV-2 infection are considered together, HTLV-associated arthropathy presents a pattern of symmetrical polyarthralgia, with large joints predominantly being affected. The presence of other joint manifestations, such as morning stiffness lasting longer than 30 min, joint effusion, and crepitations, has also been reported only in individuals with HTLV-1 infection [11].

All the patients with diffuse muscle pain were diagnosed with fibromyalgia, as previously described [17]. There is limited information regarding the clinical relationship between this rheumatological disease and HTLV infection; only one epidemiological study was found, in which fibromyalgia was diagnosed in 38% of the population with HTLV infection compared with 4.8% of the control population [40]. Concerning other chronic viral infections, such as the hepatitis C virus, there are theories that some viral infections may contribute to the etiopathogenesis of fibromyalgia. One of the theories is the sensitization of the central nervous system from exogenous toxins of the virus, which increases the production of substance P and decreases the production of serotonin, which leads to pain [41].

Patients with local muscle pain were diagnosed mainly with regional muscle pain syndromes, such as low back pain, neck pain, anserine tendonitis, trochanteric bursitis, and epicondylitis. Although there are already reports of patients infected with HTLV-1 manifesting regional pain syndromes, the etiology of this group of diseases is associated with other factors, such as work overload, sedentary lifestyles, obesity, and stress [11,42]. Notably, to our knowledge, this is the first report in the literature of low back pain recorded in a person infected with HTLV-2.

The only inflammatory autoimmune disease diagnosed in the present study was psoriatic arthritis in a patient with HTLV-1 infection. Psoriatic arthritis has a multifactorial etiology involving genetic, immunological, environmental, and infectious factors [42]. However, psoriatic arthritis is not a common clinical correlation with HTLV infection compared with other autoimmune diseases, such as rheumatoid arthritis or Sjögren’s syndrome, but it is suggested that the virus may induce an autoimmune condition to initiate the clinical onset of this pathology [26,28]. Other evidence in this study that was suggestive of autoimmunity was the presence of high ANA titers in two individuals infected with HTLV-1 but without sample reactivity for the other autoantibodies tested.

Ultimately, the main limitation of our study was the inability to include a control group of individuals not infected with HTLV, preventing a comparative evaluation of the occurrence and frequency of the clinical findings observed in the patient group. Furthermore, the selection process of individuals may have favored the attraction of individuals who already have symptoms or who are more concerned about their health and, therefore, the prevalence and spectrum of rheumatological manifestations reported in this study may not accurately reflect the broader population of individuals living with HTLV-1/2, particularly those who are asymptomatic. The results presented here have their importance in describing the spectrum of rheumatological manifestations in these patients and, in particular, in the demonstration of symptoms in individuals infected with HTLV-2. Nevertheless, further studies will be needed in order to confirm the association of HTLV-2 with rheumatological diseases.

## 5. Conclusions

Our findings confirm that HTLV-1 infection is associated with rheumatological disease manifestations and highlight the novel finding of cases of HTLV-2 infection in patients with rheumatoid arthritis symptoms.

## Figures and Tables

**Table 1:** Sociodemographic characteristics of people infected with HTLV-1/2 treated at the SAPEVH rheumatology outpatient clinic.

Characteristics	HTLV-1/2		HTLV-1		HTLV-2	
	*n*	%	*n*	%	*n*	%
**Sex**						
Female	11	73.3	9	81.8	3	75.0
Male	4	26.7	2	18.2	1	25.0
**Age**						
Mean age ± SD	53.40 ± 16.00		51 ± 17.32		60 ± 10.80	
Range	30~81		30~81		51~74	
**Race/ethnicity**						
Black	5	33.3	4	36.4	1	25.0
Brown	6	40.0	4	36.4	2	50.0
White	4	26.7	3	27.2	1	25.0
**Marital status**						
Married	7	46.7	6	54.5	1	25.0
Single	5	33.3	3	27.3	2	50.0
Divorced	1	6.7	1	9.1	0	0
Widowed	2	13.3	1	9.1	1	25.0

HTLV: human T-lymphotropic virus; SAPEVH: Service for Assistance to People Living with HTLV; SD: standard deviation.

**Table 2:** Frequency of joint manifestations in people infected with HTLV-1/2 treated at the SAPEVH rheumatology outpatient clinic by type of viral infection.

Joint Manifestations	HTLV-1 (*n* = 10)		HTLV-2 (*n* = 3)		HTLV-1/2 (*n* = 13)	
	*n*	%	*n*	%	*n*	%
**Arthralgia**	10	76.9	3	23.1	13	100
Monoarthralgia	1	10	0	0	1	7.7
Symmetrical oligoarthralgia	2	20	0	0	2	15.4
Asymmetrical oligoarthralgia	2	20	0	0	2	15.4
Symmetrical polyarthralgia	5	50	2	66.7	7	53.8
Asymmetrical polyarthralgia	0	0	1	33.3	1	7.7
**Arthritis**	2	100	0	0	2	100
Monoarthritis	1	50	0	0	1	50
Symmetrical oligoarthritis	0	0	0	0	0	0
Asymmetrical oligoarthritis	0	0	0	0	0	0
Symmetrical polyarthritis	1	50	0	0	1	50
Symmetrical polyarthritis	0	0	0	0	0	0
**Other joint manifestations**	6	66.7	3	33.3	9	100
Crepitus	5	83.3	2	66.7	7	77.8
Morning stiffness (>30 min)	1	16.4	0	0	1	11.1
Joint effusion	0	0	1	33.3	1	11.1
**Location of affected joints**	28	75.6	9	24.4	37	100
Hands	5	17.8	2	22.2	7	19
Wrists	2	7.2	0	0	2	5.4
Elbows	2	7.2	0	0	2	5.4
Shoulders	4	14.2	1	11.2	5	13.5
Feet	3	10.8	2	22.2	5	13.5
Ankles	3	10.8	0	0	3	8
Knees	5	17.8	2	22.2	7	19
Hips	4	14.2	2	22.2	6	16.2

Monoarthralgia/monoarthritis (1 affected joint); oligoarthralgia/oligoarthritis (2 to 4 affected joints); polyarthralgia/polyarthritis (5 or more affected joints); HTLV: human T-lymphotropic virus; SAPEVH: Service for Assistance to People Living with HTLV.

**Table 3:** Classification of muscle pain according to the extent and clinical characteristics of fibromyalgia in patients infected with HTLV-1/2 who were treated at the SAPEVH rheumatology outpatient clinic.

Muscle Pain	HTLV-1 (*n* = 5)		HTLV-2 (*n* = 3)		HTLV-1/2 (*n* = 8)	
	*n*	%	*n*	%	*n*	%
**Myalgia**	5	62.5	3	37.5	8	100
Localized pain	3	60	1	25	4	50
Diffuse body pain	2	40	2	75	4	50
**Fibromyalgia characteristics for diffuse body pain**	2	50	2	50	4	100
Chronic pain	2	25	2	25	4	25
Tender points	2	25	2	25	4	25
Sleep disorders	2	25	2	25	4	25
Mood disorders	2	25	2	25	4	25

Chronic pain was considered pain present for at least 3 months. To consider tender points being present, 11 or more tender points must have been present. To consider sleep disorders being present, insomnia or nonrestorative sleep must have been present. To consider mood disorders being present, excessive sadness or anxiety must have been present. HTLV: human T-lymphotropic virus; SAPEVH: Service for Assistance to People Living with HTLV.

**Table 4:** Frequency of rheumatological diseases found in people infected with HTLV-1/2 treated at the SAPEVH rheumatology outpatient clinic.

Rheumatological Diseases	HTLV-1 (*n* = 11)		HTLV-2 (*n* = 4)		HTLV-1/2 (*n* = 15)	
	*n*	%	*n*	%	*n*	%
Fibromyalgia	2	18.2	2	50	4	26.6
Psoriatic arthritis	1	9.1	0	0	1	6.6
Osteoarthritis	3	27.3	2	50	5	33.3
Epicondylitis	1	9.1	0	0	1	6.6
Low back pain	1	9.1	1	25	2	13.3
Neck pain	1	9.1	0	0	1	6.6
Chondromalacia	1	9.1	0	0	1	6.6
Carpal tunnel syndrome	2	18.2	0	0	2	13.2
Anserine tendonitis	1	9.1	0	0	1	6.6
Trochanteric bursitis	1	9.1	0	0	1	6.6

The diagnoses of rheumatological diseases were based on the clinical and laboratory criteria of the American College of Rheumatology. HTLV: human T-lymphotropic virus; SAPEVH: Service for Assistance to People Living with HTLV.

## Data Availability

The original contributions presented in this study are included in the article. Further inquiries can be directed to the corresponding author(s).
